# Prenatal Vitamin D Deficiency Induces an Early and More Severe Experimental Autoimmune Encephalomyelitis in the Second Generation

**DOI:** 10.3390/ijms130910911

**Published:** 2012-08-30

**Authors:** Diana Andrea Fernandes de Abreu, Véréna Landel, Adrian G. Barnett, John McGrath, Darryl Eyles, Francois Feron

**Affiliations:** 1 Neurobiology of Cell Interactions and Neurophysiopathology, Centre National de la Recherche Scientifique, UMR 7259, Aix Marseille Université, Marseille 13015, France; E-Mails: diana.fernandes_de_abreu@kcl.ac.uk (D.A.F.A.); verena.landel@univ-amu.fr (V.L.); 2 School of Public Health and Social Work & Institute of Health and Biomedical Innovation, Queensland University of Technology, 60 Musk Avenue, Kelvin Grove, Qld 4059, Australia; E-Mail: a.barnett@qut.edu.au; 3 Queensland Brain Institute, The University of Queensland, St Lucia, Qld 4072, Australia; E-Mails: j.mcgrath@uq.edu.au (J.M.); eyles@uq.edu.au (D.E.); 4 Queensland Centre for Mental Health Research, The Park Centre for Mental Health, Richlands, Qld 4077, Australia

**Keywords:** vitamin D experimental autoimmune encephalomyelitis, multiple sclerosis, deficiency, season of birth, transgenerational

## Abstract

In a previous study, we demonstrated that mouse adult F_1_ offspring, exposed to a vitamin D deficiency during pregnancy, developed a less severe and delayed Experimental Autoimmune Encephalomyelitis (EAE), when compared with control offspring. We then wondered whether a similar response was observed in the subsequent generation. To answer this question, we assessed F_2_ females whose F_1_ parents (males or females) were vitamin D-deprived when developing in the uterus of F_0_ females. Unexpectedly, we observed that the vitamin D deficiency affecting the F_0_ pregnant mice induced a precocious and more severe EAE in the F_2_ generation. This paradoxical finding led us to assess its implications for the epidemiology of Multiple Sclerosis (MS) in humans. Using the REFGENSEP database for MS trios (the patient and his/her parents), we collected the parents’ dates of birth and assessed a potential season of birth effect that could potentially be indicative of the vitamin D status of the pregnant grandmothers. A trend for a reduced number of births in the Fall for the parents of MS patients was observed but statistical significance was not reached. Further well powered studies are warranted to validate the latter finding.

## 1. Introduction

It has been proposed that those born in winter/spring months have an increased risk of Multiple Sclerosis (MS) [1–6]. While not all studies have confirmed this season of birth [7,8], the most well-powered study to date based on data from Northern Hemisphere countries, reported that the risk of MS was significantly higher for those born in May and lower for those born in November [9]. This season of birth effect, combined with an observed latitude gradient in incidence (higher risk at higher latitude) [10], suggest that an environmental factor, possibly vitamin D exposure, may be important in predicting MS risk.

In order to test the hypothesis that low vitamin D status during gestation is a risk factor for MS, we explored a well-described developmental vitamin D (DVD) deficiency rodent model, [11,12]. In this model, the vitamin D deficiency experienced by the offspring is restored by the 1st week of life, therefore the model is not associated with any abnormal bone development and calcium and PTH levels in the adult offspring are normal [13]. Based on findings from epidemiology, we predicted that offspring exposed to low prenatal vitamin D would be at increased risk of MS. In order to recreate such an MS-related phenotype in the mouse, we have recently used the well-described experimental autoimmune encephalomyelitis (EAE) model in DVD-deficient mice [14]. EAE induces an immune response to components of myelin and has utility to explore pathways mediating demyelination and, as an experimental platform, to screen potential therapeutic agents to treat MS. It is important to note that this model does not recreate the entire etiopathogenesis of MS, which is still poorly understood.

Contrary to our prediction, we observed that C57BL/6 mouse adult offspring exposed to DVD deficiency developed a less severe and delayed EAE, when compared with control offspring [14,15]. Curiously, DeLuca and Plum [16] have demonstrated that prolonged vitamin D deficiency, maintained for two generations, also diminished the severity and delayed the onset of EAE in B10PL mice.

**Figure 1:**
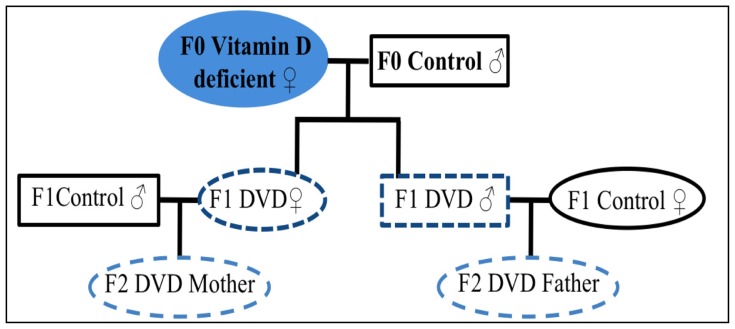
Schematic view of the experimental model. All mice, except F_0_ females, were fed with a standard vitamin D-containing mouse chow. F_0_ females were vitamin D-deprived six weeks prior mating and maintained on a deficient diet during pregnancy. F_1_ DVD-deficient offspring, females and males, were mated with control mice. Only F_2_ female offspring were subjected to Experimental Autoimmune Encephalomyelitis (EAE).

Though paradoxical, the response of the first generation to the EAE model suggests that a transient maternal hypovitaminosis D results in an altered immune response after exposure to the myelin-related protein. It remained however to be seen whether this environment-related change could be associated with any altered immune-related phenotype in the subsequent generation, as has been previously demonstrated in rats exposed to a fungicide [17]. We wondered if the second generation (F_2_) had any persisting alterations in immune function related to the vitamin D status of the pregnant F_0_ female mice. With the aim of exploring this hypothesis, we designed a new experimental model, schematized in Figure 1.

In this series of experiments, only the pregnant F_0_ female mice and their growing fetuses were vitamin D deficient. F_0_ female C57BL/6 mice were fed a vitamin D-free diet before and during pregnancy. In order to highlight potential differences between male and female germlines, we set up two groups for the F_1_ generation: DVD-deficient males were mated with control females, while DVD-deficient females were mated with control males. A vitamin D-containing diet was provided to all animals, before, during and after pregnancy. F_2_ offspring were weaned and adult females were subjected to EAE.

Paradoxically, we observed that the vitamin D deficiency affecting the F_0_ pregnant mice induced a precocious and more severe EAE in the F_2_ generation. This unexpected finding led us to assess its implications for the epidemiology of MS in humans. The question we have addressed was whether one’s parents’ season of birth (as a proxy-measure of developmental vitamin D status) could be an additional risk factor for MS patients? Using the REFGENSEP database for MS trios (the patient and his/her parents), we collected the parents’ dates of birth and assessed a potential season of birth effect that could potentially be indicative of the vitamin D status of the pregnant grandmothers.

## 2. Results and Discussion

### 2.1. A Prenatal Vitamin D Deficiency Displays a Transgenerational Effect and Induces a More Severe EAE in F_2_ Female Adult Mice

**Figure 2:**
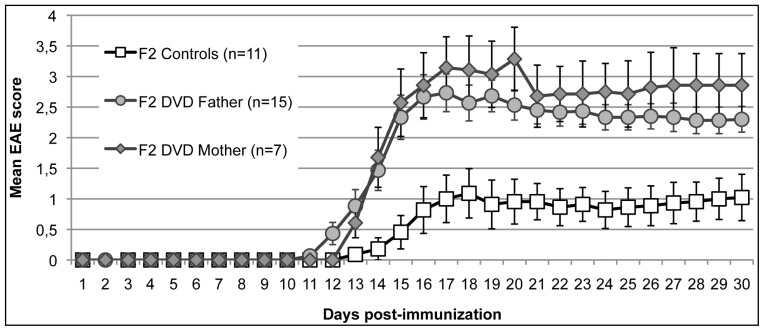
Clinical course of MOG35-55-induced EAE in F_2_ mice. F_2_ female mice born to either a DVD-deficient F_1_ Mother or a DVD-deficient F_1_ Father display an early and more severe EAE when compared with control mice.

The three tested groups developed MOG35-55-induced EAE (Figure 2). However, DVD-deficiency in the F_0_ generation significantly altered the EAE course in the F_2_ generation. Female F2 offspring from both DVD-deficient maternal and paternal sources developed a measurable EAE (*i.e.*, level 1) with a significantly earlier onset (onset day 13.7 ± 0.4 and 13.4 ± 0.5, respectively) when compared to controls (onset day 16.3 ± 0.6, F = 8.6, *p* = 0.0011). Moreover, both DVD groups displayed an increased peak in clinical score (3.1 ± 0.3 and 3.4 ± 0.5, respectively) when compared to controls (1.6 ± 0.4, *F* = 5.95, *p* = 0.0066). At Day_0_ of immunization, the mean weight of the three tested groups was similar (DVD Mother: 19.2 ± 0.5; DVD Father: 20.7 ± 0.4 g; controls: 21.2 ± 0.9).

In a previous experiment, we demonstrated that a prenatal vitamin D deficiency induces a milder and delayed EAE in the F_1_ generation [14]. In line with other studies describing a generational transmission of molecular disturbances ([18], for a recent review), we initially hypothesized that the F_2_ generation would display a phenotype similar to the F_1_ generation. However, we clearly observed a greater sensitization to the MOG immunization F_2_ females from DVD-deficient F_0_ females when compared to controls. In addition, it can be highlighted that, contrary to a human study indicating a female-associated transmission of HLA anomalies in MS families [19], no gender specific transmission was observed in our study.

This discordant behavior between that reported in DVD-deficient F_1_ offspring and the F_2_ generations is unusual and, to the best of our knowledge, has never been reported. It is not unlikely that a positive effect of a potentially deleterious environmental factor remains unnoticed. Conversely, an impaired response is easily noticeable but sometimes can only be associated to the grandparents’ and not the parents’ lifestyle, as exemplified by a seminal epidemiological study on ancestral food supply [20]. If confirmed, our findings may provide additional evidence for future studies aiming to explain generation-skipping transmission of deleterious effects.

The molecular basis of this inheritance is unclear. The role of chromatin and DNA methylation in epigenetics has been extensively studied during the past three decades. However, recent evidence supports a bigger role for RNA in gametes, including piRNAs and miRNAs that can travel between cells and silence transposable elements [18]. Among the current candidates, we can cite miR-22 that is induced by vitamin D and acts as an antiproliferative and antimigratory agent in cancer cells [21] or miR-125b that regulates the expression of human vitamin D receptor and abolishes the anti-proliferative action of calcitriol [22]. Nonetheless, to date, not a single study has yet demonstrated a piRNA- or a miRNA-associated action of vitamin D on the immune or the nervous system.

We previously demonstrated that a postnatal vitamin D supplementation reduced the severity of EAE and delayed the onset of symptoms [15]. It would be now of great interest to confirm that a similar phenotype is observed when the supplementation occurs during pregnancy. Additionally, we should perform a transgenerational study in order to assess whether the F_2_ generation is positively affected by a high dose of vitamin D delivered to the F_0_ generation.

### 2.2. Observation of a Trend for a Reduced Number of Births in the Fall for the Parents of MS Patients

**Figure 3:**
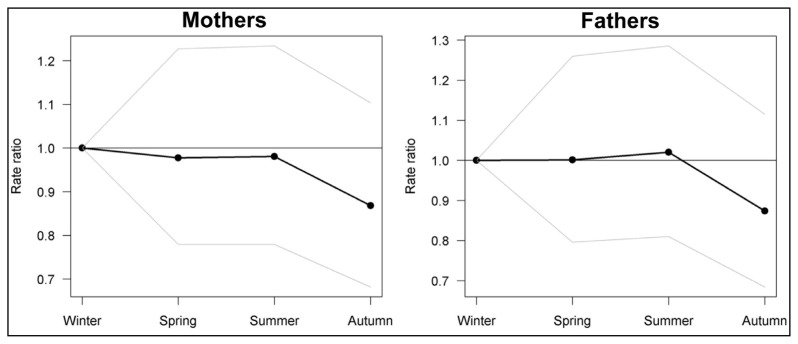
Pooled analysis of observed/expected births in parents of MS patients (*n* = 610 per group). The decreased number of births in the Fall is not statistically significant.

Parents’ dates of births were clustered into seasons as follows: Winter (December, January, February), Spring (March, April, May), Summer (June, July, August) and the Fall (September, October, November). Figure 3 indicates that the lowest risk for both groups is consistently in the Fall, although this pattern was not statistically significant (*p*-values: 0.28 mothers, 0.25 fathers).

Using the same database, we performed a case-only analysis based on children, with the cases as children born in summer and autumn, and the controls born in winter and spring. The aim of this analysis was to identify a difference in the pattern of the parents’ birthdays for MS children born in the high risk time (winter and spring—cases), compared with the low risk time (summer and autumn—controls). Our prior hypothesis was that children born in the low risk time could have had parents with higher risks, and that this might partially contributes to their MS instead of in utero exposure. The exposure variable was the circular distance from 15 October for the mother’s and father’s birthdays. The largest exposure value was π (half the circumference of a circle) for a birthday on 15 April, exactly six months away from 15 October. However, we found no evidence of any difference in the pattern of parents’ birthdays for case and control children (*p* = 0.12 and *p* = 0.89 for the mothers and fathers groups, respectively).

The current study failed to find an increased number of MS births in Spring, as suggested by our animal model. Nonetheless, the reduced number of births in the Fall, in each arm of the trio (children, fathers, mothers), requires further attention. Although not statistically significant, this finding is consistent with previous studies demonstrating a nadir of MS patients born in November [2,9]. Being born in the Fall means that, during the third trimester of pregnancy, the fetus developed in a supposedly vitamin D optimal environment. It is therefore likely that his/her immune and nervous systems benefited from vitamin D’s well-described anti-inflammatory actions [23].

## 3. Experimental Section

### 3.1. Animal Housing and Feeding

All procedures were performed according to the French law on Animal Care Guidelines. Animal Care Committee of University Aix-Marseille II approved protocols. C57Bl/6 mice (Charles River, St-Germain-sur-l’Arbresle, France) were maintained in a holding room at a constant temperature of 21 ± 2 °C and 60% relative humidity, on a 12 h light–dark cycle. Food and water were provided ad libitum. Vitamin D deficiency was achieved by: (i) feeding fertile adult F_0_ female mice with a normo-phosphatic vitamin D3-free diet supplemented with lactose and calcium (INRA, Jouy-en-Josas, France); and (ii) using UV-free lighting. Control animals (males and females) were given a standard vitamin D3-containing (1500 IU/kg) diet (INRA, Jouy-en-Josas, France). Serum vitamin D depletion was assessed six weeks later using a commercial RIA (Diasorin, Stillwater, MN, USA) for 25-hydroxyvitamin D_3_. Dams exposed to six weeks of vitamin D3 depletion exhibited a severely reduced production of 25-hydroxyvitamin D approaching the limit of detection for this assay (mean of 3 ± 0.5 ng/mL) when compared with control dams (40 ± 2.5 ng/mL). Vitamin D-deficient females were then mated with control males and kept under vitamin D3-free conditions throughout gestation. At birth, F_1_ offspring and dams were placed on standard mouse chow containing vitamin D3. In parallel, control females were mated with control males in order to obtain control offspring (control mice). DVD F_1_ offspring were weaned 28 days after birth kept on control diet and mated with control offspring. The resultant DVD-deficient and control F_2_ offspring were weaned and females, born to either DVD F_1_ males or DVD F_1_ females, were subjected to EAE at 12 weeks of age.

### 3.2. Active MOG35-55-Induced EAE

A total of thirty-three female F_2_ offspring were immunized subcutaneously with 250 μg of 35–55 MOG peptide (sequence: MEVGWYRSPFSRVVHLYRNGK, Genepep, St Jean de Védas, France), emulsified in complete Freund adjuvant (DIFCO, Lawrence, KS, USA) and supplemented with 400 μg of H37Ra Mycobacterium tuberculosis (DIFCO, Lawrence, KS, USA). One hundred nanogram of pertussis toxin was injected i.p., at Day_0_ and Day_1_ post-immunization. Pilot studies indicated that this dose of MOG peptide induced a very mild EAE in control animals. Immunized females were randomly placed in different cages. Weight and disease severity were blindly scored, once a day, according to the EAE clinical scale: 0 = no detectable sign of EAE; 1 = weakness of the tail; 2 = tail paralysis and hind limb weakness; 3 = partial paralysis of hind limbs; 4 = complete paralysis of hind limbs; 5 = complete paralysis of hind limbs with incontinence and partial or complete paralysis of forelimbs; 6 = dead [14,15].

### 3.3. Season of Birth Analysis

MS French family trios were prospectively recruited as part of a survey of MS patients identified throughout France by REFGENSEP, the French MS Genetics Group [24]. Trios were ascertained through one patient (child) per family and two parents available for typing. All patients included in the databank were examined by a neurologist, and fulfilled diagnosis criteria for definite MS. Informed consent was given by each individual participating to the study, in accordance with the Helsinki convention (1964) and French law relating to biomedical research. The whole set of 610 pairs of unrelated parents of MS patients were considered for the season of birth study. The numbers of births per month and year for the mothers and fathers were compared with the numbers for the general French population, during the same time period of 1901 to 1960.

### 3.4. Statistical Analysis

EAE data were analyzed using parametric one-way analysis of variance (ANOVA). In all analyses, *p* < 0.05 was selected as the threshold for statistical significance.

The ratio of the observed to expected number of births for the parents was analyzed using a general linear model assuming a Poisson distribution. The number of births was the dependent variable, with season as the independent variable and the log-transformed total number of births as an offset (in order to analyze the ratio of observed to expected). This analysis was made using the *R* software [25].

## 4. Conclusions

We show here for the first time that a maternal vitamin D deficiency induces inverted transgenerational effects on the immune system of offspring: the F_2_ generation is more sensitive to myelin peptide immunization while, as previously demonstrated, the F_1_ generation is less sensitive. In addition, there is no gender dimorphism. EAE symptoms in the F_2_ generation are equally increased if it is either the F_1_ mother or the F_1_ father that has been vitamin D-deprived during embryogenesis. The molecular mechanisms underlying these paradoxical immune responses are currently under investigation.

In an attempt to translate these findings to humans with MS, we looked at the parents’ seasons of birth. In line with our animal study, we predicted that parents developing in a vitamin D poor environment during the third trimester of pregnancy, in other words those born in Spring, will be at higher risk of giving birth to MS individuals. Our prediction was not validated. However, we found that parents developing in a supposedly vitamin D rich environment (*i.e.* born in the Fall) may be at lower risk of having an MS progeny. Further well powered studies are now warranted to validate this finding.

## Abbreviation

DVD: Developmental Vitamin D
EAE: Experimental Autoimmune Encephalomyelitis
MOG: Myelin Oligodendrocyte Glycoprotein
MS: Multiple Sclerosis
REFGENSEP: Reseau d’Etudes Francais GENetique sur la Sclerose En Plaques
